# Rhythmogenesis evolves as a consequence of long-term plasticity of inhibitory synapses

**DOI:** 10.1038/s41598-018-31412-7

**Published:** 2018-08-29

**Authors:** Sarit Soloduchin, Maoz Shamir

**Affiliations:** 1 Department of Physics, Faculty of Natural Sciences, Ben-Gurion University of the Negev, Be’er-Sheva, Israel; 2 Zlotowski Center for Neuroscience, Ben-Gurion University of the Negev, Be’er-Sheva, Israel; 3 Department of Physiology and Cell Biology, Faculty of Health Sciences, Ben-Gurion, University of the Negev, Be’er-Sheva, Israel

## Abstract

Brain rhythms are widely believed to reflect numerous cognitive processes. Changes in rhythmicity have been associated with pathological states. However, the mechanism underlying these rhythms remains unknown. Here, we present a theoretical analysis of the evolvement of rhythm generating capabilities in neuronal circuits. We tested the hypothesis that brain rhythms can be acquired via an intrinsic unsupervised learning process of activity dependent plasticity. Specifically, we focused on spike timing dependent plasticity (STDP) of inhibitory synapses. We detail how rhythmicity can develop via STDP under certain conditions that serve as a natural prediction of the hypothesis. We show how global features of the STDP rule govern and stabilize the resultant rhythmic activity. Finally, we demonstrate how rhythmicity is retained even in the face of synaptic variability. This study suggests a role for inhibitory plasticity that is beyond homeostatic processes.

## Introduction

Rhythmic activity has been reported to be related to a range of cognitive processes including the encoding of external stimuli, attention, learning and consolidation of memory^1–5^. In certain cases, changes in rhythmicity have been associated with pathological states^6–8^. Numerous theoretical studies have investigated various mechanisms that may produce these rhythmic behaviors. All these mechanisms assume specific parameter ranges, such as the mean synaptic strength between different neuronal populations. Deviations from the assumed parameter range results in drastic changes to rhythmic activity. However, the underlying mechanism that allows the synaptic weights, for example, to evolve into a state of rhythmic activity, and then to choose and stabilize a specific rhythm still remains enigmatic. What also remains unclear is whether this mechanism can be based on activity dependent plasticity^9,10^.

Here we consider a specific type of activity dependent plasticity known as spike timing dependent plasticity (STDP). STDP can be thought of as an extension of Hebb’s rule^11^ to the temporal domain that takes the effect of the causal relationship between pre- and post-synaptic firing on the potentiation and depression of the synapse into account. STDP has been identified in various systems in the brain, and a rich repertoire of causal relations has been described^12–22^.

Considerable theoretical efforts have been devoted to investigating the possible computational implications of STDP^23–41^. It was shown that Hebbian STDP of excitatory synapses can give rise to the emergence of response selectivity at the level of the post-synaptic neuron by inducing competition between correlated subgroups of input neurons^24,25,30^. For example, in the visual system, modeling studies have shown how spatial correlations together with STDP can develop response selectivity in the form of ocular dominance and directional selectivity^24,27,42–46^. On the other hand, Hebbian STDP of inhibitory synapses may provide a homeostatic mechanism that can balance the excitatory and inhibitory inputs to the cell^17,35,39,47,48^.

Oscillatory activity may have a strong effect on STDP as oscillations cause neurons to fire repeatedly with distinct spike timing relations. In the context of development, oscillations and repeated spatiotemporal patterns of activity may play an important role in shaping emergent neuronal connectivity maps^49,50^. The effect and possible computational role of rhythmic activity on STDP has been addressed in several studies^51–60^. However, in all of these studies the rhythmic activity was either an inherent property of the neuron or inherited via feed-forward connections from inputs that were already oscillating.

Can STDP contribute to the development of temporal structure in the neuronal response? In a recent work it was shown that STDP can contribute to synchronization in a network of interneurons oscillating in the gamma frequency^61^. It was further shown that STDP can facilitate the propagation of synchronous activity^62^. A numerical study simulating a large scale detailed thalamocortical model argued that oscillations may emerge with STDP^63^. However, the principles that underlie the emergence of oscillations with STDP remain unclear.

Here we investigated whether rhythmic behavior can emerge via a process of STDP, and if so – under what conditions, and how the features of the STDP rule govern the resultant rhythmic activity? We addressed these fundamental questions in a modelling study. We chose to study STDP dynamics using the framework of a simplified toy-model of two competing inhibitory populations with reciprocal inhibition. The choice of the toy-model was made primarily to enable complete analytical parsing. Nevertheless, it is important to note that this type of architecture has been widely used to model winner-takes-all computation and decision making^64–69^, has been observed in various brain regions^70–72^, and was recently suggested to implement a latency code readout mechanism for fast decisions^73^. Short term plasticity was also incorporated into our model in the form of firing rate adaptation in a manner that is similar to that of^74,75^ to enable a richer dynamical structure for the neuronal responses^76–78^. As we are interested in obtaining as complete analytical understanding we primarily focus on the most compact model description of reciprocal inhibition with the smallest number of parameters.

Below, we first define the dynamical model for the neuronal responses and analyze it for fixed synaptic weights. This analysis provides the phase diagram of the system, which depicts the different possible dynamical states of the network as a function of the synaptic weights. Next, we introduce STDP. STDP induces a flow along the phase diagram of the system, by allowing the synaptic weights themselves to evolve according to the plasticity rule in an activity dependent manner. This flow is then analyzed in the limit of slow learning. We show that under a broad range of parameters STDP can generate and stabilize oscillatory activity in the brain, and, that this oscillatory activity can be governed by global features of the STDP rule. Finally, we summarize our results and discuss possible outcomes and extensions to the simplified model studied here.

## Results

### The neuronal response model

We explored STDP dynamics in a model of two neuronal populations with reciprocal inhibition. The spiking activity of individual neurons in each population was modelled as an inhomogeneous Poisson process with a mean firing rate that obey the following dynamics:

1 $${\tau }_{m}{\dot{r}}_{1x}=-\,{r}_{1x}+g({I}_{1}-\frac{1}{{N}_{2}}\sum _{y=1}^{{N}_{2}}{J}_{1x,2y}{r}_{2y}-{a}_{1x})$$

2 $${\tau }_{a}{\dot{a}}_{1x}=-\,{a}_{1x}+A{r}_{1x}$$

3 $${\tau }_{m}{\dot{r}}_{2y}=-\,{r}_{2y}+g({I}_{2}-\frac{1}{{N}_{1}}\sum _{x=1}^{{N}_{1}}{J}_{2y\mathrm{,1}x}{r}_{1x}-{a}_{2y})$$

4 $${\tau }_{a}{\dot{a}}_{2y}=-{a}_{2y}+A{r}_{2y}$$

where *N*_*i*_ is the number of neurons in population *i* = 1, 2, *r*_*ix*_ is the firing rate of neuron *x* in population *i* that receives external excitatory input *I*_*i*_. For simplicity we take *I*_1_ = *I*_2_ ≡ *I*. Throughout this paper, the function *g*(*x*) will be taken to be a threshold linear function of its input, $g(x)={\lfloor x\rfloor }_{+}=x$ for *x* > 0 and 0 otherwise (see also^79^). The term *a*_*ix*_ represents the adaptation variable of neuron *x* in population *i*, and parameter *A* denotes the adaptation strength. *J*_*ix*,*jy*_ ≥ 0 is the strength of the inhibitory coupling from neuron *y* in population *j* to neuron *x* in population *i*.

Parameter *τ*_*m*_ is the membrane time constant and *τ*_*a*_ is the time constant of the adaptation. It is assumed that adaptation is a slower process than the neural response to its input, *τ*_*a*_ > *τ*_*m*_. Thus, the neuronal firing rate follows changes in its input with a time scale of *τ*_*m*_ and then adapts its rate in response to a constant input with a time scale of *τ*_*a*_ by decreasing its firing rate by a factor of 1 + *A*. We further assume, for simplicity, that the populations are relatively homogeneous. Thus, we omit the sub-indices *x* and *y* from Eqs (1–4). *r*_*i*_ represents the mean activity in population *i*, and *J*_*ij*_ the mean synaptic weight from a pre-synaptic neuron in population *j* to a post-synaptic neuron in population *i*, see Eqs (21–24) in Methods. In the limit of slow adaptation, $\epsilon \equiv {\tau }_{m}/{\tau }_{a}\to 0$, a complete analytical solution is possible; see the phase diagram section and the limit cycle calculations in Methods. Unless noted otherwise the results are given in the $\epsilon \to 0$ limit and time is measured in units of the adaptation time constant, *τ*_*a*_.

### The phase diagram

**Figure 1:**
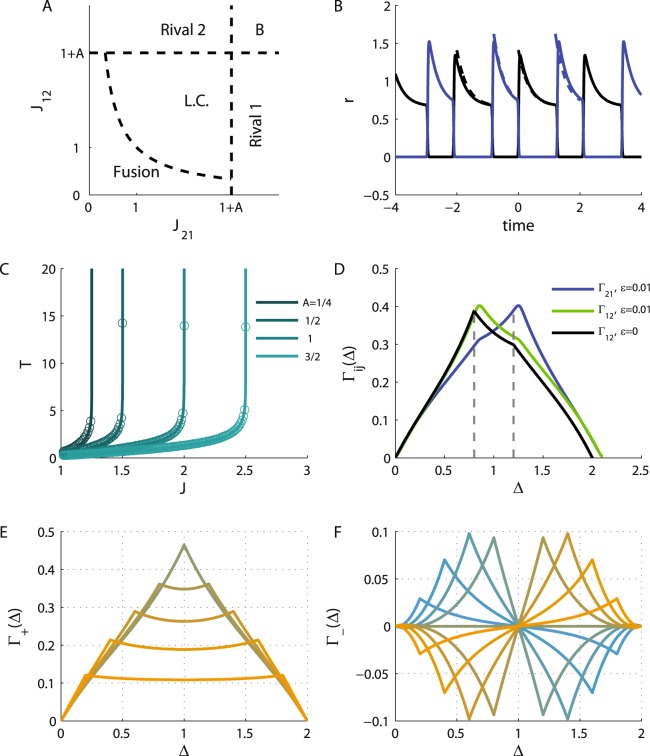
Neuronal dynamics. (**A**) The phase diagram. The regions of different types of solutions for the neuronal dynamics are depicted in the (quarter of the) plane of (non-negative) *J*_21_ and *J*_12_. (**B**). The limit cycle solution. The firing rate of populations 1 and 2 are plotted in black and blue, respectively, as a function of time (measured in units of *τ*_*a*_) in the anti-phase oscillatory solution with *T*_1_ = 1.2 and *T*_2_ = 0.8, yielding *J*_21_ ≈ 2.36 and *J*_12_ ≈ 1.87 (see Eq. (43)). In this specific example we used *I* = 2, *A* = 2, the solid lines show the solution for $\epsilon =0.01$ and the dashed depict the solution in the limit of $\epsilon \to 0$. (**C**) The oscillation period along the diagonal. The oscillation period on the diagonal is shown as a function of the reciprocal inhibition strength for different values of the adaptation strength, *A* = 0.25, 0.5, 1, 1.5 from left to right. Solid lines show the analytical relation of Eq. (44) in the $\epsilon \to 0$ limit. The circles depict the $\epsilon =0.01$ case. (**D**) The cross-correlation function. The neuronal cross-correlations Γ_12_ (green and black) and Γ_21_ (blue) are plotted as a function of the time difference, Δ (measured in units of the adaptation time constant *τ*_*a*_). The black line depicts the correlations in the $\epsilon \to 0$ limit, whereas the green and blue lines show the $\epsilon =0.01$ case. Parameters were identical to B. For the $\epsilon =0.01$ case the correlations were evaluated from the numerical solution for the dynamics. (**E**) The ‘mean cross-correlation’ function. The mean correlation, Γ_+_, in the limit of $\epsilon \to 0$, (see subsection Calculation of the cross-correlation function in Methods) is plotted as a function of Δ for *T* = 2 and different values of the *T*_1_ = *T*[0.1, 0.2, … 0.9] shown by color. Note that the plots for *T*_1_ = *x* and *T*_1_ = *T* − *x* overlap. (**F**) The ‘difference cross-correlation’. The difference in the cross-correlation, Γ_−_, in the limit of $\epsilon \to 0$, is plotted as a function of Δ for *T* = 2 and different values of the *T*_1_ = *T* × {0.1, 0.2, … 0.9} shown by color from yellow (*T*_1_ = 0.1*T*) to blue (*T*_1_ = 0.9*T*). In E and F *A* = 2 and *I* = 2 were used.

Figure 1A depicts the phase diagram of the model in the plane of *J*_12_ and *J*_21_. If the inhibition from population 1 to population 2, *J*_21_, is sufficiently strong relative to the adaptation, *J*_21_ > 1 + *A*, there exists a fixed point solution that we term *Rival 1*, in which population 1 fully suppresses population 2 (*r*_2_ = 0). Similarly, the *Rival 2* solution, in which population 2 fully suppresses population 1, exists for *J*_12_ > 1 + *A*. The *Rival* states are stable wherever they exist and may also co-exist (this bi-stable solution is denoted by B on the phase diagram); see the fixed points of the dynamics section in Methods.

For weak reciprocal inhibition, *J*_21_ < 1 + *A* and *J*_12_ < 1 + *A*, there is a solution in which both populations are active which we term the *Fusion* state. However, the *Fusion* state loses its stability for sufficiently strong inhibition, $\hat{J}\equiv \sqrt{{J}_{21}{J}_{12}} > 1+\epsilon$. Consequently, there is a region in the phase diagram in which there is no stable fixed point solution. In this region the system relaxes to a limit cycle (i.e., a periodic oscillatory solution, denoted by L.C. on the phase diagram) of anti-phase oscillation, Fig. 1B. In this case, the limit cycle solution has two phases. During phase 1, population 1 is dominant and active, *r*_1_ > 0, whereas population 2 is quiescent, *r*_2_ = 0. However, this state is not stable. Due to the adaptation, the activity of population 1 will decrease until population 2 is released from its inhibition and will further suppress population 1. During phase 2, population 2 is dominant and population 1 is quiescent. In the limit of slow adaptation, $\epsilon \to 0$, a complete solution for the limit cycle can be derived; see the limit cycle solution section in Methods.

We denote by *T*_*i*_ the dominance time of population *i*, and by *T* = *T*_1_ + *T*_2_ the period of the oscillations; see Fig. 1B. Along the diagonal of the phase diagram, ${J}_{12}={J}_{21}=\hat{J}$, the dominance times are equal, *T*_1_ = *T*_2_ = *T*/2, and the oscillation period monotonically increases from zero on the boundary of the stable *Fusion* solution, $\hat{J}=1$, to infinity on the boundary of the *Rival* solutions, $\hat{J}={J}_{12}={J}_{21}=1+A$, Fig. 1C. The dominance time of population 1, *T*_1_, diverges to infinity on the boundary of *Rival 1* state, ${\mathrm{lim}}_{{J}_{21}\to (1+A)}\,{T}_{1}=\infty$, and similarly *T*_2_, diverges on the boundary of *Rival 2* state; see Eqs (40 and 43) in the limit cycle solution section in Methods. Thus, the basic features of the oscillatory solution can be understood from the geometry of the phase diagram.

### The correlation function

One key factor that shapes STDP dynamics is the pre-post cross-correlation function. Because neuronal activities follow independent inhomogeneous Poisson processes statistics, the cross-correlation of different neurons is given by the product of their mean firing rates. Specifically, we are be interested in the temporal average of these correlations (see below). For a periodic solution we define

5 $${{\rm{\Gamma }}}_{ij}({\rm{\Delta }})\equiv {\int }_{0}^{T}\frac{dt}{T}{r}_{i}(t){r}_{j}(t+{\rm{\Delta }}).$$

Figure 1D shows the temporal average cross-correlation, Γ_*ij*_(Δ), for finite $\epsilon$ (green and blue) and in the limit of $\epsilon \to 0$ in black. Note that the main difference is the slight deviation in the oscillation period due to finite $\epsilon$, which is more important at low *T*. A detailed derivation of the cross-correlation functions appears in Methods. To analyze the STDP dynamics it is convenient to use the following quantities:

6 $${{\rm{\Gamma }}}_{+}({\rm{\Delta }})=\frac{{{\rm{\Gamma }}}_{21}({\rm{\Delta }})+{{\rm{\Gamma }}}_{12}({\rm{\Delta }})}{2}$$

7 $${{\rm{\Gamma }}}_{-}({\rm{\Delta }})={{\rm{\Gamma }}}_{21}({\rm{\Delta }})-{{\rm{\Gamma }}}_{12}({\rm{\Delta }}),$$

as shown in Fig. 1E,F, respectively, as a function of the time difference, Δ, for *T* = 2 and different values of *T*_1_ (differentiated by color). In general, Γ_±_(Δ) are periodic functions of the time difference, Δ, with a period of *T*. Γ_+_(Δ) is a positive even function of the time difference, Δ, that is symmetric with respect to *T*/2, whereas Γ_−_(Δ) is an odd function of Δ that is anti-symmetric with respect to *T*/2. Importantly, on the diagonal of the phase diagram, from symmetry, one obtains that Γ_−_(Δ) = 0.

### The STDP rule

The above analysis was carried out for fixed values of the synaptic weights, assuming that the time scales in which the synaptic weights change are much longer than the characteristic times of the neuronal population dynamics, *τ*_*m*_ and *τ*_*a*_ (see e.g.^24,29,32,36^). Next we consider the effect of STDP. We assume that initially the synaptic weights are relatively weak (i.e., near the origin of the phase diagram in the *Fusion* state) and examine how activity dependent plasticity shapes its evolution. Hence, the STDP dynamics can be thought of as a flow on the phase diagram. We are interested in understanding how the features of the STDP rule shape this flow. In particular, we aim to elucidate when this flow leads the system into the limit cycle region. Following Luz and Shamir^36^ we write the STDP rule as the sum of two processes, potentiation and depression,

8 $${\rm{\Delta }}J=\lambda ({K}_{+}({\rm{\Delta }}t)-\alpha {K}_{-}({\rm{\Delta }}t))$$

where Δ*J* is the synaptic weight difference associated with pre and post spikes with a time difference of Δ*t* = *t*_post_ − *t*_pre_. The functions *K*_±_(*t*) are the temporal kernels for the potentiation (+) and depression (−) of the STDP rule, respectively, and *α* is the relative strength of the depression. Parameter *λ* is the learning rate. We assume that the learning process occurs on a slower time scale than the adaptation. Specifically, here we focus on the family of temporally a-symmetric exponential learning rules:

9 $${K}_{\pm }(t)=\frac{1}{{\tau }_{\pm }}{e}^{\mp Ht/{\tau }_{\pm }}{\rm{\Theta }}(\,\pm \,Ht)$$

where Θ(*x*) is the Heaviside step function, and *τ*_±_ denote the characteristic time scales of the potentiation (+) and depression (−) branches of the rule. The parameter *H* = ±1 governs the nature of the learning rule, with *H* = 1 for a “Hebbian” rule (i.e., potentiating at the causal branch, when the post fires after pre, Δ*t* > 0), and *H* = −1 for the “Anti-Hebbian” STDP rule. Below we analyze the mean field approximation in the limit of *λ* → 0.

### STDP dynamics in the limit of slow learning

#### Deriving the dynamic equations

Changes to the synaptic weights following the plasticity rule of Eq. (8) in short time intervals occur as a result of either a pre or post-synaptic spike during this interval. Thus, we obtain

10 $$\begin{array}{rcl}\dot{J}(t) & = & \lambda {\rho }_{{\rm{post}}}(t){\int }_{0}^{\infty }{\rho }_{{\rm{pre}}}(t-t^{\prime} )[{K}_{+}(t^{\prime} )-\alpha {K}_{-}(t^{\prime} )]dt^{\prime} \\  &  & +\lambda {\rho }_{{\rm{pre}}}(t){\int }_{0}^{\infty }{\rho }_{{\rm{post}}}(t-t^{\prime} )[{K}_{+}(\,-\,t^{\prime} )-\alpha {K}_{-}(\,-\,t^{\prime} )]dt^{\prime} \end{array}$$

where ${\rho }_{{\rm{post}}/{\rm{pre}}}(t)={\sum }_{l}\,\delta (t-{t}_{l}^{{\rm{post}}/{\rm{pre}}})$ is the spike train of the post/pre neuron written as a sum of delta function at the neuron’s spike times ${\{{t}_{l}^{{\rm{post}}/{\rm{pre}}}\}}_{l}$. In the limit of slow learning, *λ* → 0, the right hand side of Eq. (10) can be replaced by its temporal mean, yielding (see also^24,29,32,36^),

11 $${\dot{J}}_{ij}(t)=\lambda {\int }_{-\infty }^{\infty }{{\rm{\Gamma }}}_{ij}(\,-\,t^{\prime} )[{K}_{+}(t^{\prime} )-\alpha {K}_{-}(t^{\prime} )]dt^{\prime} .$$

In regions of the phase diagram where a stable fixed point solution exists, i.e., ${r}_{i}(t)={r}_{i}^{\ast }$, the correlation function is given by the product of the time independent means, ${\rm{\Gamma }}(t)={r}_{1}^{\ast }{r}_{2}^{\ast }$, and one obtains that ${\dot{J}}_{12}={\dot{J}}_{21}$. As the firing rates are non-negative and the temporal kernels of the potentiation and depression, *K*_±_, have an integral of one, the sign of $\dot{J}$ is determined by 1 − *α*. As a corollary, the synaptic weights will flow towards the region of the limit cycle solution from initial conditions close to the origin in the phase diagram if *α* < 1. This result holds for any choice of temporal structure for the STDP rule. In particular it is independent of the Hebbianity (the value of *H*) of the STDP rule. Note that a similar condition (*α* < 1) was assumed to be the biologically relevant choice for inhibitory plasticity in^35^. Thus, initial conditions of weak synaptic coefficients (*J*_*ij*_ close to the origin) will flow towards the region of the limit cycle solution and will enter it near the diagonal, *J*_21_ = *J*_12_.

#### Order parameters of the STDP dynamics

In the region of the limit cycle the STDP dynamics do not necessarily flow in parallel to the identity line, but rather depend on the specific limit cycle solution and on the temporal structure of the STDP rule. It is convenient to formulate the STDP dynamics in terms of the mean and relative synaptic weights,

12 $${J}_{+}\equiv \frac{{J}_{21}+{J}_{12}}{2}$$

13 $${J}_{-}\equiv {J}_{21}-{J}_{12}$$

Using the above definitions, and averaging and subtracting Eq. (11) yields

14 $${\dot{J}}_{\pm }(t)=\pm \lambda {\int }_{-\infty }^{\infty }{{\rm{\Gamma }}}_{\pm }(t^{\prime} )[{K}_{+}(t^{\prime} )-\alpha {K}_{-}(t^{\prime} )]dt^{\prime} \mathrm{.}$$

For Γ_±_ see Eqs (6 and 7) and Fig. 1E,F.

On the diagonal, *J*_12_ = *J*_21_, due to the symmetry of the limit cycle solution Γ_12_(*t*) = Γ_21_(*t*), and as a result ${\dot{J}}_{-}=0$. The mean correlation, Γ_+_, on the other hand, is a positive even function of time with a period of *T*. Near the boundary of stable *Fusion*, the oscillation frequency diverges, *T* → 0. In this limit (for $\epsilon \to 0$) the limit cycle solution for the neuronal responses will approach a square wave solution (with 50% duty cycle on *J*_12_ = *J*_21_) transitioning between 0 and 2*I*/(2 + *A*) in anti-phase. The mean correlation function, Γ_+_(Δ), will approach a triangular wave starting at 0 for Δ = 0 and peaking at 2*I*^2^/(2 + *A*)^2^ for Δ = *T*/2. Consequently, for *T* → 0, the integral on the right hand side of Eq. (14) will be dominated by the DC component of Γ_+_, yielding ${\dot{J}}_{+}(t)=\lambda {I}^{2}\frac{1-\alpha }{{(2+A)}^{2}}$ in this limit. Hence, the same condition that allows the STDP dynamics to enter the limit cycle region from the *Fusion* region will also cause it flow in the positive *J*_+_ direction after entering the Limit cycle region.

#### STDP dynamics along the diagonal

Equation (14) provides two non-linear equations for *J*_+_ and for *J*_−_ that are coupled in a non trivial manner via the dependence of the correlations on the synaptic weights. However, on the diagonal of the phase diagram the situation is simplified: since ${\dot{J}}_{-}=0$ the problem is reduced to a one dimensional flow. To analyze the dynamics of *J*_+_ on the diagonal it is convenient to write it as the sum of two terms:

15 $$\frac{1}{\lambda }{\dot{J}}_{+}={\dot{J}}_{+,{\rm{pot}}}-\alpha {\dot{J}}_{+,{\rm{dep}}}$$

16 $${\dot{J}}_{+,{\rm{pot}}/{\rm{dep}}}={\int }_{-\infty }^{\infty }{{\rm{\Gamma }}}_{+}(t){K}_{+/-}(t)dt$$

**Figure 2:**
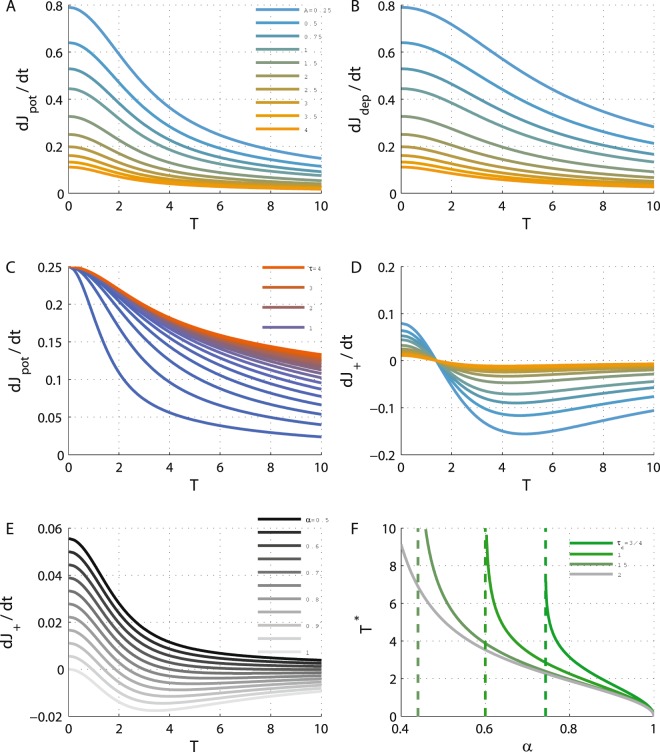
The dynamics of *J*_+_ along the diagonal. (**A**) The potentiation term, ${\dot{J}}_{+,{\rm{pot}}}$, of the mean synaptic weights, *J*_+_, Eq. (16), is shown as a function of the oscillation period along the diagonal for different values of *A* = 1/4, 1/2, 3/4, 1, 3/2, … 4 (from top at low *A* values to bottom). (**B**) The depression term, ${\dot{J}}_{+,{\rm{dep}}}$, of the mean synaptic weights, *J*_+_, Eq. (16), is shown as a function of the oscillation period along the diagonal for different values of the adaptation strength, *A* (as in A). (**C**) The effect of the STDP time constant. The potentiation term, ${\dot{J}}_{+,{\rm{pot}}}$, is shown as a function of the oscillation period along the diagonal for different values of *τ*_+_ = 1/4, 1/5, … 5, by different colors from blue (low *τ*_+_) to red. Here *A* = 2 was used. (**D**) The *J*_+_ dynamics along the diagonal. The value of ${\dot{J}}_{+}$ is shown as a function of the oscillation period along the diagonal for different values of *A* using the same values and color code as in A, using *α* = 0.9. (**E**) The effect of the relative strength of depression. The value of ${\dot{J}}_{+}$ is plotted as a function of the oscillation period along the diagonal for different values of *α* = 0.5, 0.55, … 1 from top (*α* = 0.5) to bottom (with *A* = 4). (**F**) Oscillation period at the STDP fixed point. The ‘learned’ oscillation period, T*, is shown as a function of *α* for different values of *τ*_−_ differentiated by color. The vertical dashed lines depict the value of *α*_*c*_, see calculation of *α*_*c*_ section in Methods. In all panels *I* = 2 was used, and *λ* = 1 was taken in D and E, for purposes of illustration. Unless otherwise stated, *τ*_+_ = 0.5 and *τ*_−_ = 1 used. All units of time were measured in units of *τ*_*a*_.

Figure 2A,B show ${\dot{J}}_{+,{\rm{pot}}}$ and ${\dot{J}}_{+,{\rm{dep}}}$, respectively, on the diagonal as a function of the oscillation period, *T* (note that *T* is a function of $\hat{J}$, see e.g. Fig. 1C), for different values of *A* (differentiated by color). As can be seen from the figure, ${\dot{J}}_{+,{\rm{pot}}/{\rm{dep}}}$ decreases monotonically from the value of *I*^2^/(2 + *A*)^2^ at *T* = 0 to 0 as *T* → ∞ at *J*_12_ = *J*_21_ = 1 + *A* (at the crossing to the bi-stable region). Due to the symmetry of the mean cross-correlation function, Γ_+_(*t*) = Γ_+_(−*t*), ${\dot{J}}_{+,{\rm{pot}}}$, ${\dot{J}}_{+,{\rm{dep}}}$ and ${\dot{J}}_{+}$ are independent of the Hebbianity of the STDP rule, *H*. Thus, the results of Fig. 2 hold for both Hebbian and Anti-Hebbian plasticity rules. Moreover, ${\dot{J}}_{+,{\rm{pot}}}$ and ${\dot{J}}_{+,{\rm{dep}}}$ only differ by the time constant of *K*_±_. Figure 2C shows ${\dot{J}}_{+,{\rm{pot}}}$ as a function of the oscillation period, *T*, for different values of *τ*_+_ (depicted in color). All the curves decrease monotonically to zero, albeit with a different time scale; consequently, if *τ*_+_ < *τ*_−_ then ${\dot{J}}_{+,{\rm{pot}}}\le {\dot{J}}_{+,{\rm{dep}}}$ and there is equality only at *T* = 0 (on the boundary of stable *Fusion*).

The dynamics of *J*_+_ along the diagonal are determined by the weighted sum of both ${\dot{J}}_{+,{\rm{pot}}}$ and $-\alpha {\dot{J}}_{+,{\rm{dep}}}$. ${\dot{J}}_{+}$ will be positive for *α* < 1 for small *T* - near the crossing from the *Fusion* region. For *τ*_+_ < *τ*_−_ and 1 > *α* > *α*_*c*_(*τ*_+_, *τ*_−_) (see Methods), ${\dot{J}}_{+}$ will change its sign at T*; thus, the fixed point (note ${\dot{J}}_{-}=0$ on the diagonal) at T* will be stable along the *J*_+_ direction. This scenario is illustrated in Fig. 2D that shows ${\dot{J}}_{+}$ on the diagonal as a function of *T* (for different values of *A*, depicted by color). Interestingly, for this choice of exponential kernels for the STDP rule, the fixed point does not depend on the adaptation strength, *A*. The oscillation period at the fixed point, T*, is zero for *α* = 1 and diverges as *α* approaches a critical value *α*_*c*_(*τ*_+_, *τ*_−_), Fig. 2E,F, see subsection Calculation of *α*_*c*_ in Methods. For fixed *α* ≤ 1 and *τ*_+_, T* is minimal for *τ*_−_ → ∞, increases monotonically as *τ*_−_ decreases and will diverge for a critical value *τ*_−,*c*_ < *τ*_+_ such that *α*_*c*_(*τ*_+_, *τ*_−_) = *α*. For *τ*_−_ < *τ*_−,*c*_ (and *α* ≥ 1) there will be no fixed point along the diagonal and the STDP dynamics along the diagonal will flow outside of the limit cycle region.

#### STDP dynamics away from the diagonal

The stability of the STDP fixed point requires stability in the *J*_−_ direction as well. On the diagonal ${\dot{J}}_{-}=0$. A small perturbation in the direction of *J*_−_ will affect *J*_−_ dynamics via the cross-correlation term Γ_−_(Δ), Eq. (14). The cross-correlations depend on the synaptic weight via the dominance times, *T*_1_ and *T*_2_. Hence, for a small perturbation around the diagonal, Δ*J*_−_ = *J*_−_, one obtains

17 $$\begin{array}{ccc}\frac{d{J}_{-}}{dt} & \approx  & -\lambda (\frac{d{T}_{-}}{d{J}_{-}}\,{\int }_{-{\rm{\infty }}}^{{\rm{\infty }}}\,\frac{d{{\rm{\Gamma }}}_{-}({\rm{\Delta }}|{T}_{+},{T}_{-})}{d{T}_{-}}[{K}_{+}({\rm{\Delta }})-\alpha {K}_{-}({\rm{\Delta }})]d{\rm{\Delta }}){J}_{-}\\  & = & -\lambda (\frac{d{T}_{-}}{d{J}_{-}})M{J}_{-}.\\ {T}_{+} & = & \frac{{T}_{1}+{T}_{2}}{2}\\ {T}_{-} & = & {T}_{1}-{T}_{2}\end{array}$$

The geometry of the phase diagram (Fig. 1A) reveals that increasing (decreasing) *J*_−_ results in advancing towards the *Rival 1* (*Rival 2*) region, and consequently increasing *T*_1_ (*T*_2_) and (decreasing) *T*_−_ = T_1_ − T_2_; hence, $\frac{d{T}_{-}}{d{J}_{-}} > 0$.

As above, it is convenient to define

18 $$M={M}_{{\rm{pot}}}-\alpha {M}_{{\rm{dep}}}$$

19 $${M}_{{\rm{pot}}}={\int }_{-\infty }^{\infty }\,\frac{d{{\rm{\Gamma }}}_{-}({\rm{\Delta }}|{T}_{+},{T}_{-})}{d{T}_{-}}{K}_{+}({\rm{\Delta }})d{\rm{\Delta }}$$

20 $${M}_{{\rm{dep}}}={\int }_{-\infty }^{\infty }\,\frac{d{{\rm{\Gamma }}}_{-}({\rm{\Delta }}|{T}_{+},{T}_{-})}{d{T}_{-}}{K}_{-}({\rm{\Delta }})d{\rm{\Delta }}$$

**Figure 3:**
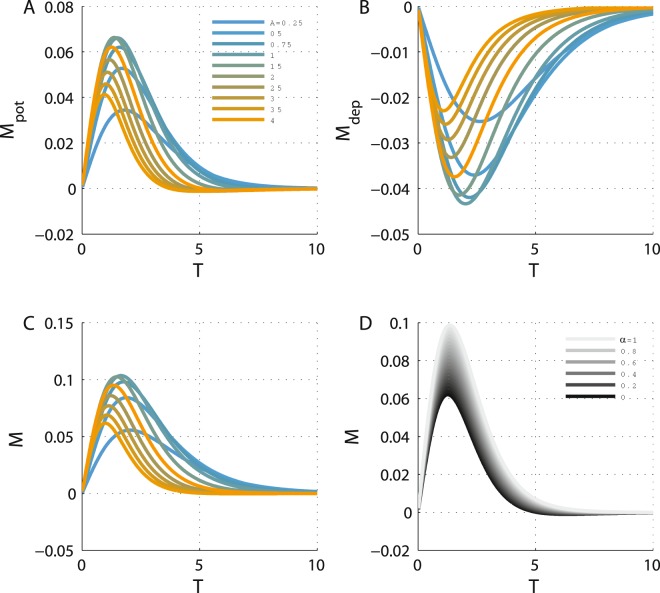
Stability in the *J*_−_ direction along the diagonal. (**A**) The value of *M*_pot_ is shown as a function of *T* along the diagonal of the phase diagram in the *Limit cycle* region for different values of *A* = 1/4, 1/2, 3/4, 1, 3/2, … 4 (from top at low *A* values to bottom). Here *τ*_+_ = 0.5 was used. All units of time were measured in units of *τ*_*a*_. (**B**) The value of *M*_dep_ is shown as a function of *T* for different values of the adaptation strength, *A* (as in (A)). Here *τ*_−_ = 1 was used. (**C**) *J*_−_ dynamics along the diagonal. The value of *M* is shown as a function of the oscillation period along the diagonal for different values of *A* (same values and color code as in A), using *α* = 0.9. (**D**). The effect of the relative strength of depression. The value of *M* is plotted as a function of the oscillation period along the diagonal for different values of *α* = 0.5, 0.55, … 1 from bottom (dark, *α* = 0) to top (light, *α* = 1), using *A* = 2.

Similar to ${\dot{J}}_{+,{\rm{pot}}/{\rm{dep}}}$, ${M}_{+,{\rm{pot}}/{\rm{dep}}}$ is also written in the form of the integral of the product of two variables; namely, the learning rule and a term that depends on the cross-correlations. However, *M*_+,pot/dep_ is not necessarily positive, as Γ_−_(Δ) and similarly $\frac{d{{\rm{\Gamma }}}_{-}}{d{T}_{-}}$ are not necessarily positive. This is illustrated in Fig. 3A,B that show *M*_pot_ and *M*_dep_, respectively, along the diagonal as a function of *T* for different values of *A* (depicted by color) for Hebbian STDP, *H* = 1. Moreover, Γ_−_(Δ) and similarly $\frac{d{{\rm{\Gamma }}}_{-}({\rm{\Delta }})}{d{T}_{-}}$ are odd functions of Δ. Consequently, $\frac{d{\dot{J}}_{-,{\rm{pot}}}}{d{T}_{-}}$ and $\frac{d{\dot{J}}_{-,{\rm{dep}}}}{d{T}_{-}}$ in Fig. 3A,B have different signs.

The value of *M* = *M*_pot_ − *αM*_dep_ along the diagonal is depicted as a function of the oscillation period, *T*, for different values of *A* (differentiated by color) and *α* (shown by gray level) in Fig. 3C,D, respectively. Here, *M* is positive, and as a result, the STDP fixed point along the diagonal will be stable with respect to fluctuations in the *J*_−_ direction for Hebbian plasticity.

**Figure 4:**
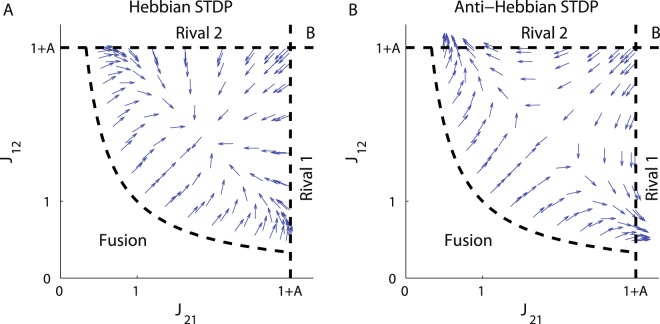
The flow on the phase diagram. The direction of the dynamic flow; i.e., the normalized vector $({\dot{J}}_{21},{\dot{J}}_{12})$, is shown in the *Limit cycle* region of the phase diagram for (**A**). Hebbian plasticity, *H* = 1 in Eq. (9), and (**B**). Anti-Hebbian plasticity, *H* = −1. The parameters used here were: *A* = 2, *τ*_+_ = 0.5 and *τ*_−_ = 1.

Finally, as Γ_−_ and similarly $\frac{d{{\rm{\Gamma }}}_{-}}{d{T}_{-}}$ are odd functions of time, switching from the Hebbian plasticity rule, *H* = 1, to Anti-Hebbian, *H* = −1, will result in a change of the sign of *M*_pot_, *M*_dep_ and of *M*. Consequently, a fixed point (on the diagonal) that is stable in the *J*_−_ direction for Hebbian plasticity will be unstable for Anti-Hebbian plasticity and vice versa. Figure 4 shows the flow induced by the STDP on the phase diagram for the (A) Hebbian and (B) Anti-Hebbian learning rules. As can be seen, the Anti-Hebbian learning rule is unable to converge to a state that allows oscillatory activity. In contrast, the Hebbian STDP generates symmetric (*T*_1_ = *T*_2_) anti-phase oscillatory activity in which the oscillation period is determined and controlled by the relative strength of the depression, *α*. This specific learning rule provides robustness with respect to the strength of adaptation, *A*. Fluctuations in *A* do not affect the period of the oscillation.

## Discussion

We examined whether rhythmic activity can emerge via an unsupervised learning process of STDP. Our main result is that under a wide range of parameters, rhythmicity can develop via STDP. Specifically, we found that to develop the capacity for rhythmic activity, the STDP rule must obey the following conditions (*i*) a bias towards potentiation, *α* < 1, will lead the system into the oscillatory region of the phase diagram, (*ii*) a longer characteristic time for depression than for potentiation, *τ*_−_ > *τ*_+_, will enable the existence of a fixed point on the diagonal that can be governed by the exact value of *α*, and (*iii*) the stability of the fixed point in the orthogonal direction is governed by the ‘Hebbianity’ of the plasticity rule.

Using the framework of a simplified toy-model, Magnasco and colleagues studied the computational implications of neuronal plasticity in recurrent networks^80^. It was claimed that Anti-Hebbian plasticity rules drive the network into a state of near criticality. This raises the question of why we did not find traces of criticality in our model. The explanation has to do with the differences between our models. The most significant is the temporal structure of the learning rule, which together with the neuronal cross-correlations is the driving force of STDP dynamics. In their work, Magnasco and colleagues used an instantaneous plasticity rule. Consequently, only correlations at a zero time difference, which are inherently symmetric, contributed to their learning dynamics. As a result, only the symmetric part of their synaptic connectivity pattern was affected by the dynamics. In contrast, in our model both *J*_+_ and *J*_−_, which denote the symmetric and anti-symmetric parts of the connectivity pattern - respectively, evolve with time. When one allows for a non-trivial STDP rule, much richer dynamical behaviors can develop^60^. Nevertheless, it is important to emphasize we do not claim that that Hebbian but not Anti-Hebbian plasticity will induce rhythmogenesis. We found that due to inherent symmetry if the Hebbian STDP fails to yield rhythmogenesis then the Anti-Hebbian can, and vice-versa.

### Control of rhythmic activity

STDP may also provide a mechanism for selecting and stabilizing oscillations; for example, the oscillation frequency can be governed and manipulated by the relative strength of the depression, *α*, or changes in the time constants of the STDP rule, *τ*_±_, see Fig. 2F. Disruption of the STDP rule may result in changes to the learned oscillation frequency.

### Simplifying assumptions

The analysis of STDP dynamics in recurrent networks is challenging. To facilitate the analysis we used the framework of a simplified model for the neuronal responses and made several simplifying assumptions. We assumed a separation of three time scales ${\tau }_{m}\ll {\tau }_{a}\ll {\lambda }^{-1}$. The separation of the neuronal time constant from that of the adaptation enabled us to obtain an analytic expression for the temporal correlations that drive the STDP dynamics. The assumption that long term synaptic plasticity occurs on a longer time scale allowed us to consider STDP dynamics as a flow on the phase diagram. Numerous studies have employed phase diagram description to depict the possible dynamical states of the network as a function of various parameters. Our approach to STDP dynamics adds another layer to this description.

**Figure 5:**
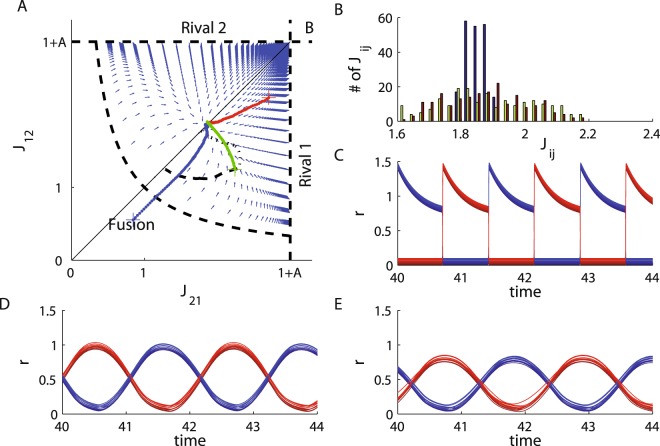
Numerical simulation of STDP dynamics. We solved the STDP dynamics numerically, Eq. (11), with *N*_1_ = *N*_2_ = 10, *α* = 0.9, *τ*_+_ = 0.5 and *τ*_−_ = 1. The cross-correlation functions were evaluated numerically using the separation of time scales. For each update step of the synaptic weights, the cross-correlations were evaluated by numerically solving the 2(*N*_1_ + *N*_2_) dynamics of the neuronal firing rates, Eqs (1–4), with fixed values for the synaptic weights with *I* = 2, *A* = 2 and $\epsilon =0.001$. (**A**) Trajectories of the order parameters, ${J}_{ij}=\frac{1}{{N}_{1}{N}_{2}}\,{\sum }_{x,y}\,{J}_{ix,jy}$, for five simulations are plotted on the phase diagram and the flow chart. The red, green and blue traces depict the learning dynamics of the same model with $\epsilon =0.001$ from different initial conditions (marked by +). The dashed black curve depicts the learning dynamics of the order parameters with $\epsilon =0.2$. The dotted black curve depicts the learning dynamics of the order parameters for a model with a local inhibition term ${J}_{{\rm{loc}}}=0.5$, and $\epsilon =0.2$. The vector field shows the STDP flow for $\epsilon =0$ calculated using the analytic expressions for the correlations, subsection Calculation of the cross-correlation function in Methods. (**B**) Synaptic weight distribution for the three examples with $\epsilon =0.001$ in A (red, green and blue), differentiated by color. (**C**) Neuronal dynamics at the STDP fixed point for the slow adaptation case, $\epsilon =0.001$. The firing rates of the *N*_1_ population 1 neurons (red traces) and *N*_2_ population 2 neurons (blue traces) in arbitrary units are shown as a function of time. Since the firing rates of different neurons from the same population overlapped, we shifted them vertically for purposes of visualization. For the three different initial conditions (with $\epsilon =0.001$) illustrated in A the oscillation period was *T* = 1.433, *T* = 1.432, and *T* = 1.436, and all units of time are measured in units of *τ*_*a*_. (**D**) Neuronal dynamics with $\epsilon =0.2$ at the STDP fixed point. The firing rates of the *N*_1_ population 1 neurons (red traces) and *N*_2_ population 2 neurons (blue traces) in arbitrary units are shown as a function of time. The firing rates of different neurons were shifted vertically for purposes of visualization. The oscillation period was *T* = 2.165 in units of *τ*_*a*_. (**E**) Neuronal dynamics with local inhibition, *J*_loc_ = 0.5, and $\epsilon =0.2$ at the STDP fixed point. The firing rates of the *N*_1_ population 1 neurons (red traces) and *N*_2_ population 2 neurons (blue traces) in arbitrary units are shown as a function of time, see subsection Neuronal dynamics with local inhibition term in Methods. The firing rates of different neurons were shifted vertically for purposes of visualization. The oscillation period was *T* = 2.17 in units of *τ*_*a*_.

Figure 5 shows numerical solutions for the STDP dynamics. The vector field depicts our analytic solution to the STDP dynamics using exact expressions for the cross-correlations (see calculation of the cross-correlation function in Methods) in the limit of $\epsilon =0$. The red, green and blue traces show the results of numerically simulating the STDP dynamic with a neuronal model with a small but finite $\epsilon =0.001$. As can be seen from the figure, the numerical results with $\epsilon =0.001$ closely adhere to the analytically calculated flow and converge to the same fixed point. The dashed black line depicts the numerical results of simulating the model with $\epsilon =0.2$. Taking $\epsilon =0.2$ affects the temporal pattern of the oscillations, Fig. 5D (compare with Fig. 5C for $\epsilon =0.001$); mainly no population is ever fully suppressed. This affects the cross-correlation function, which in turn will modify the flow along the phase diagram. As a result, the STDP dynamics will converge to a different fixed point. Nevertheless, the STDP dynamics still converges to a state of anti-phase oscillations, Fig. 5C. Thus, although quantitatively the results are different, similar qualitative behavior is obtained. The limit of small $\epsilon$ enabled us to obtain complete analytical expressions for the cross-correlations.

The interplay between short and long term plasticity processes deserves consideration. Oscillations would not be possible in this model without short term plasticity; here, adaptation. Thus, short term plasticity plays a major role in shaping the temporal structure of the neuronal cross-correlations, Γ_*ij*_(*t*) that drive the STDP dynamics, which in turn, may or may not converge to a state that allows this oscillatory behavior. It is interesting to note that short term plasticity, specifically the value of *A*, affects and shapes the phase diagram. Decreasing the value of *A* to zero, for example, will shrink the region of oscillatory activity to zero and rhythmic activity will no longer be possible.

The reflection of the flow on the phase diagram with respect to the diagonal when reflecting the STDP rule with respect to time stems from the inherent symmetry of the cross-correlation function which drives the dynamics (Γ_*ij*_(Δ) = Γ_*ji*_(−Δ)); hence, it is general and holds regardless of the choice of model. Certain other assumptions can easily be relaxed. For example, we assumed symmetry between the two competing populations. However, using the (threshold) linearity of our model one can easily rescale the neuronal responses to allow for different inputs and adaptation strengths. On the other hand, the independence of the fixed point, T*, on the adaptation strength, *A*, is specific to this model and for the choice of an exponentially decaying STDP rule.

### Choice of network architecture

A central assumption in this study was the choice of (a reciprocal inhibition) architecture. Because it is not possible to analyze a system with an undefined architecture some choice had to be made. The specific choice of architecture was made to obtain a model that could be fully analyzed. However, the choice of architecture (including the short-term-plasticity mechanism) shapes the phase diagram, allows for the different regions of dynamical solutions (fixed points, In/Out of/Anti -phase oscillations, etc.) and determines the cross-correlations. Additionally, under certain conditions, propagation delays may also have a major effect on the computational outcome of the STDP dynamics^81^. For example, The dotted line in Fig. 5A shows the numerical results of simulating the STDP dynamics in a neuronal model that also incorporates within population inhibition, see section neuronal dynamics with local inhibition term in Methods. The STDP dynamics with local inhibition follows a different flow on the phase diagram and converges to a different point. Nevertheless, the STDP dynamics converges to a state of anti-phase oscillations, Fig. 5E. Incorporating a local inhibition term will not only modify the flow on the phase diagram, but will also change the phase diagram itself. Consequently, the effect of the network architecture on STDP dynamics should not be underestimated. Because this effect is highly non-linear, one cannot generalize these results to other architectures in a straightforward manner. Nevertheless, the approach delineated here; namely, studying the induced flow on the phase diagram of the system, can be applied to other models in the limit of a slow learning rate.

### Robustness to synaptic variability

Yet another central simplifying assumption we made throughout our analysis was that the population-mean firing rates and the mean synaptic weights were representative of single neuron firing rates and single synaptic weights. This allowed us to explore a model with reduced dimensionality (the phase diagram was analyzed in 2 dimensions instead of 2*N*_1_*N*_2_ dimensions) and only to study the dynamics of the global order parameters such as the *effective* (or mean) couplings between the two populations. However, it is not at all obvious that the mean synaptic weight is indeed a good representative of the synaptic weight distribution, since for instance, the neuronal populations may break down into sub-clusters. Figure 5A shows that even when individual synapses are free to potentiate and depress independently, the mean weights follow the predicted flow on the phase diagram. Moreover, the mean synaptic weights remain representative of the individual weights that are distributed around them, Fig. 5B. Nevertheless, the distribution around the mean weights is not trivial. Consequently, different neurons may receive different levels of inhibition and miss-tuning of the oscillation frequency might occur. Figure 5C shows that the firing rates of different neurons (vertically shifted) in the two populations (differentiated by color) are identical in spite of the synaptic weights distribution. Moreover, even though synaptic weight distribution is different in the three examples shown (differentiated by color), the oscillation period is almost identical. Thus, functionality, in terms of obtaining a specific oscillation frequency, is retained even in the face of synaptic variability. What is the source of this remarkable outcome? We believe that this results from the fact that the STDP dynamics (e.g. Eq. (11)) only depend on the synaptic weights via the cross-correlations, which in turn, are determined by the oscillation period and dominance times. Thus, the fixed point of the STDP dynamics itself is determined by the oscillation period due to the activity dependence of the plasticity rule. On the other hand, to obtain this rhythmic activity it was also essential to have an architecture with two distinct inhibitory populations.

## Methods

### Phase diagram and limit cycle calculations

#### The fixed points of the dynamics

From Eqs (1–4) we obtain the dynamics of the mean firing rates in each population

21 $$\epsilon {\dot{r}}_{1}=-\,{r}_{1}+{\lfloor {I}_{1}-{J}_{12}{r}_{2}-{a}_{1}\rfloor }_{+}$$

22 $${\dot{a}}_{1}=-\,{a}_{1}+A{r}_{1}$$

23 $$\epsilon {\dot{r}}_{2}=-\,{r}_{2}+{\lfloor {I}_{2}-{J}_{21}{r}_{1}-{a}_{2}\rfloor }_{+}$$

24 $${\dot{a}}_{2}=-\,{a}_{2}+A{r}_{2}$$

We also rescaled time and hereafter measure time in units of the adaptation time constant. We distinguish two types of fixed points: *Rival* states, in which one population fully suppresses the other, and *Fusion*, in which both populations are active.

#### The Rival states

The *Rival-1* solution assumes ${r}_{1}^{\ast } > 0$ and ${r}_{2}^{\ast }=0$, yielding ${r}_{1}^{\ast }=I/(1+A)$, ${a}_{1}^{\ast }=IA/(1+A)$ and ${a}_{2}^{\ast }={r}_{2}^{\ast }=0$. The existence condition for this solution is that the net input to population 2, *I* − *J*_21_*r*_1_ − *a*_2_ is non-positive, at the fixed point, *J*_21_ ≥ 1 + *A*. This solution is always stable where it exists.

#### The Fusion state

The *Fusion* solution assumes ${r}_{1}^{\ast } > 0$ and ${r}_{2}^{\ast } > 0$, yielding

25 $$(\begin{array}{c}{r}_{1}^{\ast }\\ {r}_{2}^{\ast }\end{array})=\frac{I}{{\mathrm{(1}+A)}^{2}-{\hat{J}}^{2}}(\begin{array}{c}1+A-{J}_{12}\\ 1+A-{J}_{21}\end{array})$$

26 $${a}_{i}^{\ast }=A{r}_{i}^{\ast },\,(i=1,2)$$

where $\hat{J}=\sqrt{{J}_{12}{J}_{21}}$. The existence of the *Fusion* solution requires the inputs of both populations to be non-negative. For ${\hat{J}}^{2} < {(1-A)}^{2}$ the existence condition requires *J*_12_ ≤ 1 + *A* and *J*_21_ ≤ 1 + *A* (bottom left square in the phase diagram, Fig. 1A, where no *Rival* solution exists). By contrast, for ${\hat{J}}^{2} > {(1-A)}^{2}$ the existence condition requires *J*_12_ ≥ 1 + *A* and *J*_21_ ≥ 1 + *A* (the region in the phase diagram where both *Rival* solutions exist). However, the *Fusion* state is not always stable. By performing standard stability analysis around the *Fusion* fixed point we expand the dynamics around the fixed point to a leading order in the fluctuations

27 $$\frac{d}{dt}\,(\begin{array}{c}\delta {r}_{1}\\ \delta {a}_{1}\\ \delta {r}_{2}\\ \delta {a}_{2}\end{array})=-[\begin{array}{cccc}1 & 1 & {J}_{12} & 0\\ -\epsilon A & \varepsilon  & 0 & 0\\ {J}_{21} & 0 & 1 & 1\\ 0 & 0 & -A\epsilon  & \varepsilon \end{array}]\,(\begin{array}{c}\delta {r}_{1}\\ \delta {a}_{1}\\ \delta {r}_{2}\\ \delta {a}_{2}\end{array})$$

where $\delta x\equiv x-{x}^{\ast }$, yielding the four eigenvalues for the stability matrix:

28 $$2{\lambda }_{{\mp }^{1},{\pm }^{2}}=-\,\mathrm{(1}+\epsilon {\mp }^{1}\hat{J}){\pm }^{2}\sqrt{{\mathrm{(1}+\epsilon {\mp }^{1}\hat{J})}^{2}-4\epsilon \mathrm{(1}+A{\mp }^{1}\hat{J})}$$

The sum of the pair of eigenvalues ${\lambda }_{{+}^{1},{\pm }^{2}}$ is $-\hat{J}-(1+\epsilon ) < 0$ and their product is $\epsilon (1+A{+}^{1}\hat{J}) > 0$; hence, these eigenvalues are always stable. On the other hand, for the pair of eigenvalues ${\lambda }_{{-}^{1},{\pm }^{2}}$ the sum is $+\hat{J}-(1+\epsilon )$, which is negative if and only if inhibition is sufficiently weak, $\hat{J} < 1+\epsilon$ (in that case their product will also be positive, assuming $\epsilon$ is small). Thus, the *Fusion* state loses its stability when reciprocal inhibition becomes sufficiently strong, $\hat{J} > 1+\epsilon$.

#### The limit cycle solution

In the region of the phase diagram where no stable fixed point exists the network dynamics relaxes to anti-phase oscillations. Below we provide a detailed solution for the limit cycle in the limit of $\epsilon \to 0$. The limit cycle is solved using the anti-phase oscillations ansatz. First the neuronal dynamics is solved for each phase, where the dynamics are linear. This provides a piecewise solution with several parameters to be determined. Then we apply two sets of constraints: periodicity and transition.

Assuming the anti-phase oscillations ansatz we separate the cycle into two phases. During *phase-1* population 1 is dominant and fully suppresses population 2, for times *t* ∈ (0, *T*_1_). In the limit of slow adaptation, $\epsilon \to 0$, dynamics during *phase-1* are given by:

29 $${r}_{1}=I-{a}_{1}\,(t\in \mathrm{(0,}{T}_{1}))$$

30 $${\dot{a}}_{1}=-\,\mathrm{(1}+A){a}_{1}+AI$$

31 $${r}_{2}=0$$

32 $${\dot{a}}_{2}=-\,{a}_{2}$$

where we measure time in units of *τ*_*a*_. Eqs (29–32) can be easily solved, yielding

33 $${a}_{1}(t)={a}_{1}\mathrm{(0)}{e}^{-\mathrm{[1}+A]t}+\frac{IA}{1+A}\mathrm{(1}-{e}^{-\mathrm{[1}+A]t}),\,(t\in \mathrm{(0,}\,{T}_{1}))$$

34 $${a}_{2}(t)={a}_{2}(0){e}^{-t}$$

Similarly, during *phase-2*, when population 2 is dominant and fully suppresses population 1, $t=t^{\prime} +{T}_{1}\in$ $({T}_{1},{T}_{1}+{T}_{2})$, we obtain

35 $${a}_{1}(t^{\prime} +{T}_{1})={a}_{1}({T}_{1}){e}^{-t^{\prime} },\,(t^{\prime} \in \mathrm{(0,}{T}_{2}))$$

36 $${a}_{2}(t^{\prime} +{T}_{1})={a}_{2}({T}_{1}){e}^{-\mathrm{[1}+A]t^{\prime} }+\frac{IA}{1+A}\mathrm{(1}-{e}^{-\mathrm{[1}+A]t^{\prime} })$$

Continuity of the adaptation variables, *a*_*i*_, dictates that, for example, the initial conditions of Eq. (36), *a*_2_(*T*_1_), will be given from Eq. (34) by ${a}_{2}({T}_{1})={a}_{2}(0){e}^{-{T}_{1}}$. We now need to determine four parameters: *a*_1_(0), *a*_2_(0), *T*_1_ and *T*_2_. These parameters are determined by two sets of constraints. One is periodicity, namely

37 $${a}_{i}(0)={a}_{i}({T}_{1}+{T}_{2}),\,i\in \{1,2\}$$

yielding,

38 $${a}_{1}\mathrm{(0)}=I\frac{A}{1+A}F({T}_{1},{T}_{2})$$

39 $${a}_{2}({T}_{1})=I\frac{A}{1+A}F({T}_{2},{T}_{1})$$

40 $$F(x,y)=\frac{\mathrm{(1}-{e}^{-\mathrm{[1}+A]x}){e}^{-y}}{1-{e}^{-\mathrm{[1}+A]x-y}}$$

The second set of constraints is given by the transition conditions. Specifically, the transition time from *phase-1* to *phase-2* at *T*_1_ is not arbitrary; rather, *T*_1_ is a special point in time in which population 2 is released from being fully suppressed, such that the net input to population 2 changes its sign from negative to positive; thus,

41 $$0=I-{J}_{21}{r}_{1}({T}_{1})-{a}_{2}({T}_{1})$$

42 $$0=I-{J}_{12}{r}_{2}(0)-{a}_{1}(0)$$

which provides implicit equations for the dominance times, *T*_1_ and *T*_2_,

43 $${J}_{ij}=\frac{1-\frac{A}{1+A}F({T}_{i},{T}_{j})}{1-\frac{A}{1+A}F({T}_{j},{T}_{i}){e}^{{T}_{i}}},\,(i,j)\in \mathrm{\{(1,2),}\,\mathrm{(2,1)\}}$$

Using Eq. (43), and taking the limit of *T*_1_ → ∞, we obtain *J*_21_ → 1 + *A*. Thus, the dominance time of population *i*, *T*_*i*_, diverges on the boundary of *Rival-i*. Taking the limit of *T*_1_, *T*_2_ → 0 such that *T*_1_/*T*_2_ = *β*, yields ${J}_{21}\to \frac{1+\beta (1+A)}{1+A+\beta }$ and from symmetry ${J}_{12}\to \frac{1+1\,/\,\beta (1+A)}{1+A+1\,/\,\beta }$, which obeys *J*_12_*J*_21_ → 1; hence, the limit of the zero oscillation period is obtained on the boundary of stable *Fusion* (note that these calculations were done for $\epsilon \to 0$).

On the diagonal, ${J}_{12}={J}_{21}\equiv \hat{J}$, dominance times are equal, *T*_1_ = *T*_2_ = *T*/2,

44 $$\hat{J}=\frac{1-\frac{A}{1+A}F(T/2,T/2)}{1-\frac{A}{1+A}F(T/2,T/2){e}^{T/2}}$$

Consequently, the oscillation period, *T*, increases monotonically along the diagonal of the phase-diagram from zero at the transition to *Fusion* ($\hat{J}=1$) to infinity at the transition to the *Rival* states ($\hat{J}=1+A$).

### Calculation of the cross-correlation function

Calculation of the (temporally averaged) cross-correlation function, Eq. (5), is done using the analytical solution for the neuronal responses in the limit of slow adaptation, $\epsilon \to 0$. These correlations arise from co-fluctuation of the firing rates of the neurons and affect the STDP dynamics via their overlap with the STDP rule; thus, the relevant timescales are determined by the temporal structure of the STDP rule, Eq. (9). When the system relaxes to a fixed point solution, ${r}_{i}(t)={r}_{i}^{\ast }$ (*i* = 1, 2), the cross-correlations are constant in time,

45 $${{\rm{\Gamma }}}_{ij}(t)={r}_{i}^{\ast }{r}_{j}^{\ast }$$

Thus, correlations will be zero in the *Rival* states; hence, there will be no STDP. In the *Fusion* state the cross-correlations will be symmetric, Γ_12_(*t*) = Γ_21_(*t*). As a result, the STDP dynamics for *J*_12_ and *J*_21_ will be identical and the flow will be in the uniform direction, parallel to the diagonal line.

At the *Limit cycle* we use the analytical solution, Eqs (33–40), to calculate the cross-correlations in a straightforward manner. For ${\rm{\Delta }}\in [0,\,{\rm{\min }}\,\{{T}_{1},{T}_{2}\}]$ we obtain

46 $${{\rm{\Gamma }}}_{21}({\rm{\Delta }})=\frac{{I}^{2}}{T{(1+A)}^{2}}({G}_{0}+{G}_{1}+{G}_{2}+{G}_{3})$$

47 $${G}_{0}={\rm{\Delta }}$$

48 $${G}_{1}=\frac{A}{1+A}C({T}_{1},{T}_{2})\,(1-{e}^{-\mathrm{[1}+A]{\rm{\Delta }}})$$

49 $${G}_{2}=\frac{A}{1+A}C({T}_{2},{T}_{1})\,({e}^{\mathrm{[1}+A]{\rm{\Delta }}}-1){e}^{-\mathrm{[1}+A]{T}_{2}}$$

50 $${G}_{3}=\frac{{A}^{2}}{\mathrm{2(1}+A)}C({T}_{1},{T}_{2})C({T}_{2},{T}_{1})\,({e}^{\mathrm{2[1}+A]{\rm{\Delta }}}-1){e}^{-\mathrm{[1}+A]({T}_{2}+{\rm{\Delta }})}$$

where

51 $$C(x,y)=1-F(x,y)=\frac{{e}^{y}-1}{{e}^{y}-{e}^{-\mathrm{[1}+A]x}}$$

For Δ > min{*T*_1_, *T*_2_}, assuming without loss of generality that *T*_1_ ≥ *T*_2_

52 $${{\rm{\Gamma }}}_{21}({\rm{\Delta }})=\frac{{I}^{2}}{T{(1+A)}^{2}}({H}_{0}+{H}_{1}+{H}_{2}+{H}_{3})$$

53 $${H}_{0}={T}_{2}$$

54 $${H}_{1}=\frac{A}{1+A}C({T}_{1},{T}_{2})\,({e}^{\mathrm{[1}+A]{T}_{2}}-1){e}^{-\mathrm{[1}+A]{\rm{\Delta }}}$$

55 $${H}_{2}=\frac{A}{1+A}C({T}_{2},{T}_{1})\,(1-{e}^{-\mathrm{[1}+A]{T}_{2}})$$

56 $${H}_{3}=\frac{{A}^{2}}{\mathrm{2(1}+A)}C({T}_{1},{T}_{2})C({T}_{2},{T}_{1})\,({e}^{\mathrm{2[1}+A]{T}_{2}}-1){e}^{-\mathrm{[1}+A]({T}_{2}+{\rm{\Delta }})}$$

Along the diagonal, on the edge of the stable *Fusion* state region, *T* → 0, the cross-correlation will resemble a triangular chainsaw function (in the $\epsilon \to 0$ limit) with period *T* and peak 2*I*^2^/(2 + *A*)^2^. Consequently, as *T* goes to zero, the overlap between the cross-correlation function and the STDP rule will be governed by the DC component, yielding

57 $$\mathop{\mathrm{lim}}\limits_{T\to 0}\,{\dot{J}}_{+}=(1-\alpha ){(\frac{I}{2+A})}^{2}$$

The above expressions for the cross-correlations were given in terms of the dominance times, {*T*_*i*_} instead of the effective couplings *J*_*ij*_. The translation to the synaptic weights from the dominance times is possible by Eq. (43). However, because we were interested in studying the ability to learn and stabilize a specific oscillatory activity, it was more convenient to think about the dynamics in terms of the dominance times. Similarly, to consider stability with respect to the *J*_−_ direction we utilized the derivative of Γ_−_ = Γ_21_ − Γ_12_ with respect to *T*_−_ = *T*_1_ − *T*_2_. On the diagonal, ${T}_{1}={T}_{2}\equiv \bar{T}$

58 $$\frac{d{{\rm{\Gamma }}}_{-}}{d{T}_{-}}({\rm{\Delta }})=\frac{{I}^{2}}{T{(1+A)}^{2}}\frac{AC(\bar{T},\bar{T})}{1+A}({I}_{1}+{I}_{2}+{I}_{3})$$

59 $${I}_{0}=\frac{{e}^{\bar{T}}-\mathrm{(1}-A){e}^{-\mathrm{[1}+A]\bar{T}}}{{e}^{\bar{T}}-{e}^{-\mathrm{[1}+A]\bar{T}}}-\frac{{e}^{\bar{T}}}{{e}^{\bar{T}}-1}$$

60 $${I}_{1}={I}_{0}\mathrm{(1}-{e}^{-\mathrm{[1}+A]{\rm{\Delta }}})$$

61 $${I}_{2}={e}^{-\mathrm{[1}+A]\bar{T}}\mathrm{(1}+A-{I}_{0})\,({e}^{\mathrm{[1}+A]{\rm{\Delta }}}-\mathrm{1)}$$

62 $${I}_{3}=A\mathrm{(1}+A)C(\bar{T},\bar{T}){e}^{-\mathrm{[1}+A]\bar{T}}\,\sinh \,\mathrm{([1}+A]{\rm{\Delta }})$$

### Calculation of *α*_*c*_

On the diagonal *T*_1_ = *T*_2_ = *T*/2, in the limit of slow oscillations, *T* → ∞, one obtains

63 $${{\rm{\Gamma }}}_{+}({\rm{\Delta }})=\frac{{I}^{2}}{T{\mathrm{(1}+A)}^{2}}({\rm{\Delta }}+\frac{A}{1+A}(1-{e}^{-\mathrm{[1}+A]{\rm{\Delta }}}))\mathrm{.}$$

Using Eq. (63) yields

64 $${\dot{J}}_{+,{\rm{pot}}/{\rm{dep}}}=\frac{{I}^{2}}{T{\mathrm{(1}+A)}^{2}}N({\tau }_{\pm }),\,(T\to \infty )$$

65 $$N(x)=\frac{A}{1+A}+x-\frac{A}{1+A}\frac{1}{x\mathrm{[1}+A]+1}$$

Hence, if *α* is less than a critical value *α*_*c*_ = *N*(*τ*_+_)/*N*(*τ*_−_), then ${\dot{J}}_{+}$ will always be positive (along the diagonal). On the other hand, if *α* is larger than *α*_*c*_ then ${\dot{J}}_{+}$ will be negative for sufficiently large *T*, and a fixed point will exist if *α* < 1.

### Neuronal dynamics with a local inhibition term

In Fig. 5 we also show results of simulating the STDP dynamics in a model that includes a local, within population, inhibition. To this end we replace Eqs (1–4) with

66 $${\tau }_{m}{\dot{r}}_{1x}=-\,{r}_{1x}+g({I}_{1}-\frac{1}{{N}_{2}}\,\sum _{y\mathrm{=1}}^{{N}_{2}}\,{J}_{1x\mathrm{,2}y}{r}_{2y}-\frac{1}{{N}_{1}}\,\sum _{x\mathrm{=1}}^{{N}_{1}}\,{J}_{{\rm{loc}}}{r}_{1x}-{a}_{1x})$$

67 $${\tau }_{a}{\dot{a}}_{1x}=-\,{a}_{1x}+A{r}_{1x}$$

68 $${\tau }_{m}{\dot{r}}_{2y}=-\,{r}_{2y}+g\,({I}_{2}-\frac{1}{{N}_{1}}\,\sum _{x\mathrm{=1}}^{{N}_{1}}\,{J}_{2y\mathrm{,1}x}{r}_{1x}-\frac{1}{{N}_{2}}\,\sum _{y\mathrm{=1}}^{{N}_{2}}\,{J}_{{\rm{loc}}}{r}_{2y}-{a}_{2y})$$

69 $${\tau }_{a}{\dot{a}}_{2y}=-\,{a}_{2y}+A{r}_{2y}$$
